# Factors related to earthquake preparedness of households based on social-cognitive theory constructs: A systematic review

**DOI:** 10.3389/fpubh.2023.987418

**Published:** 2023-02-16

**Authors:** Esmat Rezabeigi Davarani, Mahmood Nekoei-Moghadam, Narges Khanjani, Abedin Iranpour, Mohammadreza Chashmyazdan, Hojjat Farahmandnia

**Affiliations:** ^1^Health in Disasters and Emergencies Research Center, Institute for Futures Studies in Health, Kerman University of Medical Sciences, Kerman, Iran; ^2^Environmental Health Engineering Research Center, Kerman University of Medical Sciences, Kerman, Iran; ^3^HIV/STI Surveillance Research Center, WHO Collaborating Center for HIV Surveillance, Institute for Futures Studies in Health, Kerman University of Medical Sciences, Kerman, Iran; ^4^Department of Medical Librarianship and Information Science, Kerman University of Medical Sciences, Kerman, Iran

**Keywords:** earthquake, preparedness, family, households, social-cognitive theory

## Abstract

**Background:**

Earthquakes cause many casualties worldwide. Taking preventive measures and improving community preparedness is critical to reducing earthquake damage. The social cognitive theory explains how individual and environmental factors cause behavior. This review was conducted to identify the social cognitive theory structures, in research on the preparedness of households against earthquakes.

**Materials and methods:**

This systematic review was performed based on the Preferred Reporting Items for Systematic Reviews and Meta-Analyses (PRISMA) guidelines. A search was conducted from January 1, 2000, to October 30, 2021 in Web of Science, Scopus, PubMed, and Google Scholar. Studies were selected based on inclusion and exclusion criteria. The initial search yielded 9,225 articles, and finally, 18 articles were selected. Articles were assessed using the Strengthening the Reporting of Observational Studies in Epidemiology (STROBE) checklist.

**Results:**

Eighteen articles about disaster preparedness behaviors based on the socio-cognitive constructs were identified and reviewed. The essential constructs used in the reviewed studies included self-efficacy, collective efficacy, knowledge, outcome expectations, social support, and normative beliefs.

**Conclusion:**

By identifying the dominant structures that have been used in studies related to the preparedness of households against earthquakes, researchers can implement appropriate and more cost-effective interventions by focusing on improving suitable structures.

## Introduction

The 21st century has witnessed an upward trend in natural hazards, increased casualties, and high economic loss (1, 2). Among natural disasters, earthquakes are important due to their unique characteristics, such as unpredictability, high destructive power, and high human casualties (3). Globally, 142.9 million people are at risk of earthquakes (4). The World Health Organization (WHO) reported that over the past century, 1,150 fatal earthquakes have occurred in 75 countries worldwide (5). Earthquakes yearly cause more than 10,000 deaths, most of which occur in developing countries (6).

The most efficient way to reduce earthquake losses is to design durable buildings (7). However, even in the safest structures, there is a potential for death and injury if non-structural vulnerability mitigation measures are not applied (8–10). Damage after an earthquake due to the interruption of essential services can be widespread and can negatively affect people's lives, work, and regular social processes (9, 11). Therefore, preventive measures and improving community preparedness are crucial to reducing earthquake damage (12).

Preparedness means activities and actions to ensure an effective response to hazards to minimize human and property damage (13–16). Essential measures in the preparation phase include acquiring knowledge and skills, planning to reduce the effects of hazards, providing emergency equipment and supplies, and emergency protective measures (17–20). Based on several studies conducted in Iran (21, 22), China (23), Australia, and New Zealand (24), most households were not prepared to deal with hazards.

Preparedness is affected by various demographic, behavioral, environmental, social, cognitive, economic, physical, and cultural factors (17, 21, 25, 26). Behavioral and social science theories provide a platform to understand why people engage in high-risk or protective behaviors (27). Behavior change theories and models have also been used in research on disaster risk management (12, 21, 22, 28–32).

Social cognitive theory is one of the most influential theories used in predicting behavior. This theory attempts to explain human behavior based on three pillars that are related to each other (1). Personal cognitive factors (self-efficacy, collective efficiency, outcome expectations, and knowledge), (2) socio-environmental factors (observational learning, normative beliefs, social support, barriers, and opportunities), (3) and behavioral factors (behavioral skills, intentions, reinforcement, and punishment) (33).

Since social cognitive theory has many structures, it is often not possible to study all of these structures at once. The diversity of constructs has caused researchers to choose a specific construct for evaluation, based on the type of behavior under study (34).

Considering that the current research is part of a sequential exploratory study, it was necessary to identify the structures used in related studies to design interview questions for future research. By identifying the structures that have received more attention in studies related to the preparedness of households against earthquakes, it is possible to design more appropriate tools and implement more cost-effective interventions.

Therefore, this systematic review was conducted to identify the constructs of the social cognitive theory in research related to household preparedness against earthquakes.

## Materials and methods

This systematic review was a part of a consecutive exploratory study, to design and validate a tool (interview questions) to measure the factors affecting the preparedness of households against earthquakes based on the social cognitive theory. The Preferred Reporting Items for Systematic Reviews and Meta-Analyses (PRISMA) guidelines were used. A search was conducted from January 1, 2000, to October 30, 2021, on Web of Science, Scopus, PubMed, and Google Scholar.

### Search strategy

The search strategy was developed based on keywords related to the research topic. A set of keywords was selected based on previous studies and Medical Subject Headings (MeSH). We used four groups of keywords: (a) social-cognitive theory, social cognitive model, and cognitive-social theory constructs, (b) risk, disaster, emergency, hazard, catastrophe, crisis, and earthquakes, (c) preparedness, readiness, mitigation, behavior, protective action, and preventive behaviors (d) household, family, citizen, population, resident, inhabitant, and public. These keywords were combined using the operators of the mentioned databases. The search strategy and key terms were as follows: (“social-cognitive theory” OR “social cognitive model” OR self-efficacy OR “collective efficacy” OR “outcome expectations” OR “observational learning” OR “normative beliefs” OR “social support” OR “barriers and opportunities” OR “behavioral skills” OR “reinforcement and punishment”) AND (risk^*^ OR disaster^*^ OR emergenc^*^ OR hazard^*^ OR catastroph^*^ OR crisis OR earthquake^*^) AND (prepar^*^ OR readiness OR mitigation OR behavior^*^ “protective action” OR “preventive behavior”) AND (household^*^ OR family OR citizen^*^ OR population OR resident^*^ OR inhabitant^*^ OR public). These searches were performed in abstracts, keywords, and titles. Furthermore, the reference list of published studies was also searched for relevant articles. Only English articles were included.

### Data collection

**Figure 1:**
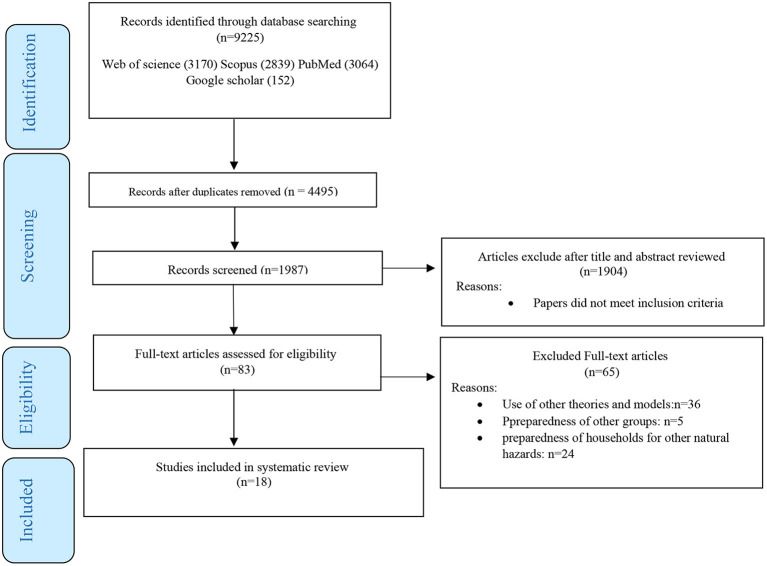
Flow diagram showing a selection of articles reviewed.

The articles from the initial search were imported into EndNote software. After removing duplicate and unrelated titles, the remaining titles, abstracts, and the full text of the articles were screened by the first author (ER) and the second author (HS). Discussions about article selection were resolved through discussion, and the relevant articles were selected. The selection process for this review is shown in Figure 1.

The initial search yielded 9,225 articles, of which 4,730 duplicate titles and 2,508 unrelated titles were deleted. The abstracts of the remaining 1,987 titles were reviewed, and 83 related articles were selected. In the next step, 65 articles were excluded. The reasons for exclusion were that these articles used other health behavior change theories, did not examine household preparedness for earthquakes and did it for other organizations and groups, or examined household preparedness for other natural hazards. Eventually, 17 articles remained. Later one study was added through a search of the reference lists of the retrieved articles (Figure 1). The data of the selected articles were extracted based on a pre-designed form by the first and second authors.

### Inclusion and exclusion criteria

Inclusion criteria were original quantitative articles about social-cognitive theory constructs related to earthquake preparedness of households. Articles in which the title was about disaster preparedness, but inside the article, the focus was on earthquakes were selected. However, articles that focused on other natural hazards, such as floods, climate change, etc., were excluded. Review studies, abstracts of papers presented at conferences, letters to the editor, editorials, and dissertations were not included.

### Quality assessment

The quality of the included literature was assessed independently by two reviewers (ER and MN–M). For qualification assessment of the articles, the Strengthening the Reporting of Observational Studies in Epidemiology (STROBE) checklist was applied (26). The checklist consisted of 22 questions. A “yes” answer would receive 1 point, and a “no, or unspecified” would receive 0 points.

## Results

Eighteen articles on disaster preparedness behaviors based on socio-cognitive theory constructs were identified and reviewed. The essential constructs used in the reviewed studies included self-efficacy, collective efficacy, knowledge, outcome expectations, social support, and normative beliefs.

The constructs of the social-cognitive theory, including self-efficacy, collective efficacy, knowledge, outcome expectations, social support, and normative beliefs in studies, had been mentioned in respectively 9, 5, 5, 4, 3, and 1 articles. None of the articles used all of the constructs together.

In two articles, the study group was vulnerable households (elderly and disabled). In one article, the study examined the staff's household preparedness level. In 15 articles, an adult or head of household participated in the study. The characteristics of each of the selected studies are presented in Table 1.

**Table 1:** Summary of studies related to earthquake preparedness behaviors of households based on the structures of the social-cognitive theory.

**References, location**	**Structures of social-cognitive theory and other variables**	**Sample size, targeted population**	**Key outcomes**
Ning et al. (23), China	Knowledge, attitudes, risk perception, self-efficacy, emergency preparedness behaviors	2,506 households. From each household, one adult who was over 16 years	The respondents' attitudes toward emergency preparedness had the strongest association with emergency preparedness behaviors. Attitudes were associated with self-efficacy, knowledge, and risk perception. Association between self-efficacy and emergency preparedness behaviors was statistically significant
Wang et al. (35), China	Self-efficacy, place attachment, disaster preparedness	2,181 households. From each household, one adult who was over 18 years	Self-efficacy and place attachment were correlated to the overall preparedness indicator. The respondents with disaster experience, older age, males, and Communist Party of China (CPC) members with higher education levels and annual household incomes tended to have significantly higher levels of disaster preparedness
Han et al. (36), China	Trust, social support, risk perception, preparedness, preventive measures to reduce the risk of earthquakes	415 households. The head of each household or the available adults within the household	Trust in the high-level government; trust in local government, disaster impact, being a male, and being married were positively correlated with the likelihood of respondents reporting they felt prepared. In contrast, informal support, trust in outside helpers, and owning more land were negatively associated with the likelihood of reporting that they felt prepared. All the other variables' effects were insignificant
Yong and Lemyre (37), Canada	Risk perception, social capital (social networks, social support, trust, volunteering), disaster preparedness behaviors	1,089 residents	Community-level social capital indicators were meaningful predictors of individuals' preparedness behaviors. Social capital was an essential ingredient in effective disaster preparedness
Adams et al. (38), United States	Self-efficacy, response efficacy, community advantage, disaster preparedness	4,700 people with disabilities, residents aged 18 and above	Self-efficacy significantly mediated the relation between self-rated health and disaster preparedness. Living in a community with more significant advantages, particularly with more advantaged social and housing attributes, reduced the negative association between poor self-rated health and preparedness
Hong et al. (39), Taiwan	Self-efficacy, quality of life, trust in government, disaster preparedness behavior	1,682 residents aged 18 and above	When self-efficacy (SE) was high, the positive relation between Quality of life (QoL) and preparedness behavior (PB) increased. The positive relation between trust in government (GT) and PB increased when SE was high. When SE was high, the mediating effect of GT on the relation between QoL and PB increased
Ranjbar et al. (40), Iran	Societal and environmental factors (trust, empowerment, community participation, collective efficacy, outcome expectancies), earthquake preparedness behaviors (real behavior, perceived readiness, and the intention to prepare)	369 residents 18 years old and above	Social trust was the most critical predictor of preparedness behavior, intention to be prepared and perceived preparedness. The averages for social trust and the other dimensions of preparedness, namely, the actual behavior and perceived preparedness, were generally less than the expected average
Adhikari et al. (41), Nepal	Community participation, collective efficacy, trust, empowerment, and behavioral intentions	306 households	Individual risk beliefs included risk appraisal (perceived probability and severity) and coping appraisal, including self-efficacy, response efficiency, and response cost. Also, community factors (community participation, collective efficacy) and institutional factors (trust, empowerment) were predicted by the intention to prepare behavior
Kelly and Ronan (24), Australia and New Zealand	Outcome expectancy, responsibility, education, collective efficacy, participation, trust, distrust, confidence, empowerment, intentions to prepare and preparedness behaviors	291 residents 18 years old and above	Intentions to prepare were not predicted by positive outcome expectancy, participation, collective efficacy, empowerment, education, responsibility, confidence, and trust. Personal responsibility and negative outcome expectancy had a significant unique effect on preparedness
Armaş et al. (42), Romania	Perceived self-efficacy, locus of control, risk perception, trust in institutions, perceived earthquake preparedness	1,376 households. In each household, an over 18-year-old	Worry consistently correlates with stress vulnerability, non-self-efficacy, and an external locus of control. Those more prepared for earthquakes scored higher on self-efficacy and lower on stress vulnerabilities
Adams et al. (43), United States	Interpersonal communication, personal responsibility, self-efficacy, outcome efficacy, knowledge, disaster preparedness	2,052 individuals who were registered for the Shakeout campaign	The Community-Oriented cluster, involved in the drill and other interpersonal activities, including attending disaster-planning meetings, was positively associated with interpersonal communication, self-efficacy, outcome efficacy, and knowledge about disaster preparedness. The Interactive and Games cluster, which participated in the drill and two online earthquake preparedness games, was positively associated with all five social cognitive factors studied
Kim and Zakour (44), United States	Social support, community participation, community trust, emergency preparedness and resource preparedness	719 adults aged 55 years and older	Individuals with higher levels of social support and connections to community organizations were more prepared for disaster-related emergencies. Higher income and higher informal support were related to a higher level of resources for disaster preparedness
Thomas et al. (45), United States	Knowledge, preparedness beliefs, risk perception, self-efficacy, household preparedness	439 employees of the Center for Disease Control and Prevention	Significant differences in reported preparedness behaviors were observed between knowledge levels. Preparedness and self-efficacy beliefs were associated with the emergency kit and written plan preparedness. Participants reporting preparedness knowledge and social connectedness were more likely to be prepared for disasters
Xu et al. (46), China	Knowledge, risk perception, attitudes, self- efficacy, emergency preparedness	2,686 households. From each household, one adult who was over 18 years	Females, higher household income, previous experience with an emergency, higher levels of emergency knowledge, risk awareness, self-efficacy, and positive attitudes were significant predictors of emergency preparedness
Paton et al. (25), Japan and New Zealand	Outcome expectancies, community participation, collective efficacy, empowerment, trust, intention to prepare	506 residents (251 from Japan and 255 from New Zealand)	In Japan, community participation was a strong predictor of collective efficiency. Outcome expectations (individual factor) predicted earthquake preparedness behavior in both societies. Community participation, collective efficiency, and trust in government significantly affected the intention to prepare. Collective efficiency was a positive predictor of empowerment, and empowerment was a positive predictor of trust in government
McIvor et al. (47), Australia and New Zealand	Outcome expectancy, general trust, collective efficacy, community participation, empowerment, behavioral intention	520 residents (264 from Australia and 256 from New Zealand)	Community participation was a positive predictor of behavioral intention and empowerment. Negative outcome expectations were negative predictors of behavioral intention, and positive outcome expectations were positive predictors of behavioral intention, collective efficiency, and empowerment. Collective efficiency predicted empowerment, and empowerment predicted general trust
McIvor and Paton (48), New Zealand	Intentions to prepare, outcome expectancies, action coping, attitudes, subjective norms, intention to seek information	156 adult residents	Positive subjective norms had no direct influence on intentions to prepare but had an indirect influence mediated by outcome expectancies. Positive subjective norms regarding hazard preparedness increased individuals' outcome expectancies
Paton et al. (49), New Zealand	Risk perception, anxiety, critical awareness, intentions to prepare, self-efficacy, outcome expectancy, intention to seek information, action coping, preparation	Phase one = 660 household phase two = 640 household	Critical awareness, risk perception, and earthquake anxiety were predictors of outcome expectations. Outcome expectations predicted self-efficacy, intention to prepare, and action coping. Critical awareness, outcome expectations, and action coping were predictors of intention to prepare. Self-efficacy and critical awareness were predictors of intention to seek information. Intentions to prepare were a strong predictor of earthquake preparedness behavior

The quality of the articles varied from 15 to 22 (Table 2).

**Table 2:** Quality of the final extracted articles using strengthening the reporting of observational studies in epidemiology (STROBE).

**Article**	**1**	**2**	**3**	**4**	**5**	**6**	**7**	**8**	**9**	**10**	**11**	**12**	**13**	**14**	**15**	**16**	**17**	**18**
Title and abstract	*	*	*	*	*	*	*	*	*	*	*	*	–	*	*	*	*	*
**Introduction**																		
Background/rationale	*	*	*	*	*	*	*	*	*	*	*	*	*	*	*	*	*	*
Objectives	*	*	–	*	–	–	*	*	*	*	*	*	–	*	*	*	*	*
**Methods**																		
Study design	*	*	*	*	*	*	*	*	*	*	*	*	*	*	*	*	*	*
Setting	*	*	*	*	*	*	*	*	*	*	*	*	*	*	*	*	*	*
Participants	*	*	*	*	*	*	*	*	*	*	*	*	*	*	*	*	*	*
Variables	*	*	*	*	*	*	*	*	*	*	*	*	*	*	*	*	*	*
Data sources/measurement	*	*	*	*	*	*	*	*	*	*	*	*	*	*	*	*	*	*
Bias	–	*	–	–	–	–	*	–	*	–	–	–	–	*	–	–	–	–
Study size	*	*	*	*	*	*	*	*	*	*	*	*	*	*	*	*	–	*
Quantitative variables	*	*	*	*	*	*	*	*	*	*	*	*	*	*	*	*	*	*
Statistical methods	*	*	*	*	*	–	–	*	–	*	*	*	*	*	*	*	*	*
**Results**																		
Participants	*	*	*	*	*	*	*	–	*	*	*	*	*	*	–	–	–	–
Descriptive data	*	*	*	*	*	*	–	*	*	*	*	*	*	*	*	*	*	*
Outcome data	*	*	*	*	*	*	*	*	*	*	*	*	*	*	*	*	*	*
Main results	*	*	*	*	*	*	*	*	*	*	*	*	*	*	*	*	*	*
Other analyses	*	*	–	–	–	–	–	–	–	–	–	–	*	–	–	–	–	–
**Discussion**																		
Key results	*	*	*	*	*	*	*	*	*	–	*	*	*	*	*	*	*	*
Limitations	*	*	*	–	*	–	*	–	*	*	*	*	*	*	–	–	*	–
Interpretation	*	*	*	*	*	–	*	–	*	–	*	*	*	*	*	–	–	–
Generalizability	*	*	*	*	*	*	*	*	*	*	*	*	*	*	*	*	*	*
**Other information**																		
Funding	*	*	*	–	*	–	*	–	–	–	–	*	–	*	–	–	–	–
STROBE Grade	21	22	19	18	19	15	19	16	19	17	19	20	18	21	17	16	16	16

* = yes; – = No.

## Discussion

The essential constructs used in the reviewed studies included self-efficacy, outcome expectations, social support, collective efficacy, normative beliefs, and knowledge.

### Self-efficacy

In the reviewed articles, self-efficacy was one of the most important structures used in research on earthquake preparedness. Bandura considers self-efficacy to be a person's judgment of his or her ability to perform a particular action (27). Studies showed that the higher a person's self-efficacy, the more intention there is for preventive measures and disaster preparedness (23, 35, 38, 39, 41–43, 45, 46, 49). People are more likely to take precautionary measures and disaster preparedness behaviors when they believe they can do it. In studies based on other theories and behavior change models, people with higher self-efficacy performed more preventive actions and disaster preparedness behaviors (22, 31, 41, 50–52). According to a study by Newnham in Hong Kong (53), people with higher self-efficacy had fewer evacuation barriers during disasters. The results of Cong et al. from the United States (54) showed that lack of self-efficacy was one of the barriers to disaster preparedness behavior. In Janis et al.'s study in the Philippines (55), people with higher self-efficacy were more prepared for typhoons.

People are more likely to engage in disaster-prevention behaviors if they are confident in their ability to engage in disaster-prevention behaviors. In order to increase disaster preparedness, intentions, and behaviors, it is necessary to increase people's belief in their abilities.

### Outcome expectations

Another construct of the social-cognitive theory used in the reviewed studies was outcome expectations. Outcome expectations anticipate possible outcomes that will result from preparedness measures (33). According to the studies, the higher the positive outcome expectations in people, the more the intention and behavior of disaster preparedness, and the higher the negative outcome expectations, the less the intention and behavior of preparedness (24, 47–49, 56). Also, the results of the reviewed studies showed that collective efficiency (47) and self-efficacy (49) were higher in societies where positive outcome expectations were reported in more people. If people are confident that their actions will have positive consequences, they will take preventive measures and improve disaster preparedness. However, if they find their efforts futile and feel helpless in the face of disasters, they will not take action to prepare themselves for disasters.

### Social support

Social support is another construct of the social-cognitive theory used in studies of disaster preparedness behaviors. According to the results of studies, people with higher levels of formal and informal social support and more connections with the community and organizations were more prepared for disaster-related emergencies (37, 38, 44, 45). According to a study by Babcicky and Seebauer (57), people who received more social support in flood-prone areas in Austria had more self-efficacy. In a study conducted by Mideksa et al. (58) in the Philippines, students receiving higher social support from family, peers, and school were better prepared for disasters. In Permana's study in Indonesia (59), higher social support increased community self-efficacy in coping with the tsunamis.

But, contrary to the findings of the mentioned studies, the study conducted by Han et al. (36) in China showed that individuals who reported more social support had lower preparedness. In a study by Yu et al. (60) in earthquake-prone villages in China, the more informal social support was received, the less preparedness was reported.

According to Bandura (61), individuals, as social beings, tend to rely on other members of society to resolve issues and improve their wellbeing. Households receiving more support from relatives and authorities may feel more empowered to take preventative measures to reduce the risk of disasters. On the other hand, a strong sense of belonging to a community and receiving more social support may reduce the feeling of concern about natural hazards and mitigation.

### Collective efficacy

Another construct used in these studies was collective efficacy. According to the social-cognitive theory, collective efficacy is the assurance of individuals that their joint efforts will bring social change (62). Compared to self-efficacy, collective efficacy beliefs are related to group tasks, group members' shared efforts and thoughts, and group progress (33). According to the results of the studies, in societies where higher collective efficacy and greater community participation were reported, the intentions and behaviors of disaster preparedness were higher (25, 31, 40, 41, 47). Studies have shown that people participating in more social events were more prepared for disasters (24, 40). In Florida, a study by Mash et al. (63) showed that the higher the collective efficacy, the more people were prepared after the hurricane. According to the study conducted by Fay-Ramirez et al. (64), respondents who were most affected by storms and floods, reported lower levels of collective efficiency before the disaster. In the study of Martins et al. (65), in New York City households that were politically active or were integrated into community networks were more likely to engage in all types of preparedness efforts.

The more extensive the social network before a disaster and the greater the social connections in the community, the less the adverse effects of a disaster. In communities where disaster risk reduction behaviors are compatible with the local community's culture, disasters cause minor damage, so planners must empower the community and involve people in social activities.

According to the results of the reviewed studies, the higher the social trust and trust in the government and authorities, the more disaster preparedness behavior was reported (25, 36, 37, 39–41, 47). While, in the study of Armaş et al. (42), those who felt less prepared were more likely to trust various institutions (government, NGOs, fire departments, media, etc.). Excessive trust in responsible authorities and organizations may make people feel less responsible, and a false sense of security may prevent them from taking precautionary measures and preparedness behaviors. On the other hand, high trust will likely improve communication between local authorities and residents and increase disaster preparedness.

### Normative beliefs

One of the constructs of the social-cognitive theory is normative beliefs. According to the results of the reviewed studies, in McIver and Patton's (48) study, people with positive subjective norms had more intentions for disaster preparedness measures. The studies based on other theories also showed that people with higher subjective norms had more prepared intentions and behavior against disasters (21, 30, 31, 66). According to Nurjana's study in Indonesia (67), the higher the subjective norms, the greater the attitude toward preparedness and preparedness behaviors against disasters increased as well. In Ong et al.'s study in the Philippines (68), subjective norm was one of the key factors that increased people's intention to prepare for an earthquake. These studies show that if there is more interpersonal and social communication and people are influenced by relatives and other important people in the community, the intention and behavior of preparedness will increase. Recognition of these beliefs may assist policymakers and executives in developing a better understanding of the origins of preparedness behaviors.

### Knowledge

According to the results of the studies, people with a higher level of knowledge had stronger beliefs, intentions, and preparedness behavior; and lack of awareness and knowledge was mentioned as one of the most important reasons for poor preparedness for emergencies (23, 43, 45, 46). Heinkel et al.'s study in Myanmar (69) shows that increasing household knowledge can improve preparedness. Wu et al. 's study in China (70) concluded that the more substantial the disaster knowledge, the better the disaster preparedness.

In Wu et al.'s (70) study, based on survey data of residents in the affected areas of Wenchuan and Lushan, residents had strong knowledge about disasters. But the residents' knowledge was weak before the earthquake.

Based on the results of reviewed studies, people who participated in the training programs or other family members had greater self-efficacy, knowledge, and preparedness (23, 24, 42). Amini et al., in Iran (12) showed that educational interventions improve the preparedness behavior of households against earthquakes.

Therefore, to improve the preparedness behavior of households against earthquakes, it is necessary to design and implement appropriate training programs based on structures that are strong predictors of intention and behavior.

### Preparedness

Studies have shown that despite global efforts to reduce disaster risk, global preparedness intentions and behaviors are unsatisfactory despite the experience of natural hazards in these populations (35, 36, 40, 45, 46). However, in most studies that reported the level of preparedness of the population under study, most households were not sufficiently prepared for disasters or did not intend to take measures related to preparedness (23–25, 35, 36, 40, 42, 44, 46, 71). Chen et al.'s study in China (72) shows that only 9.9% of households were well prepared for emergencies, 53.6% did not know what to do and 31.6% did not want to think about it. Results of a study by Martins et al. (65), indicate that the levels of household preparedness in New York City at the time of the storm were modest. The reason for the difference in the level of preparedness of households against disasters in different regions may be due to the difference in the socio-economic level of households, the experience of a destructive hazards, the perception of risk, as well as the efforts and planning of the government to improve the level of people's preparedness and reduce the risk of disasters.

In a post-Taiwan earthquake study, people had high intentions to engage in earthquake preparedness behavior, but still lacked preparedness measures (51). It is likely that after experiencing a dangerous hazard, residents' awareness and risk perception will increase, and people will be more willing to prepare for potential future hazards. Failure to prepare for disaster may be due to a lack of knowledge and skills in that population despite high intentions.

### Demographic factors

Based on the results of the studies, in several studies, older people (24, 35, 43, 45, 46) and in some studies, younger people (39, 42) had more reasonable beliefs, intentions, and disaster preparedness behaviors. Older people may report more preparedness and intentions due to more experience, better risk perception, religious beliefs, and responsibility toward younger people. However, limited mobility and disability at older ages can negatively affect appropriate behavior when hazards occur, or the feeling of being close to the end of life can reduce their motivation for disaster preparedness. Younger people are more likely to have better beliefs, intentions, and preparedness behaviors than older people, because of their higher levels of education and better economic, social, and physical status.

In most studies, men reported better belief, intent, and behavior in the face of disasters (24, 35, 36, 39, 42, 45), and in some articles, women reported better intent and preparedness (43, 46). Differences in the findings of different studies can be due to cultural, social, and economic differences in different regions. Another reason may be that the head of the household has more responsibility than other family members.

People with higher levels of education (35, 39, 42, 45) and incomes (24, 35, 42–46) were more prepared for disasters.

According to the results of the reviewed studies, people with physical disabilities and their families (38), and ethnic, racial, and political minority groups (36, 43) had lower beliefs, intentions, knowledge, and disaster preparedness behaviors.

## Limitations

This article only reviewed studies published in English, and may therefore be subject to language bias.

## Conclusion

The constructs of self-efficacy, collective efficacy, knowledge, outcome expectations, social support, and normative beliefs were used more frequently in studies related to earthquake preparedness behaviors of households. Designing and implementing interventions focusing on these structures can improve preventive behaviors and the preparedness of households against earthquakes.

## Data availability statement

The original contributions presented in the study are included in the article/supplementary material, further inquiries can be directed to the corresponding author.

## Author contributions

ER conceived the concept and design of the study. AI conducted the survey. HF and MC were involved in data analysis and manuscript writing. MN-M and NK supervised the study and critically reviewed the manuscript. All authors read reviewed the final manuscript.
